# The modulated low-temperature structure of malayaite, CaSnOSiO_4_

**DOI:** 10.1107/S2052520620003807

**Published:** 2020-04-16

**Authors:** Thomas Malcherek, Bianca Paulenz, Michael Fischer, Carsten Paulmann

**Affiliations:** a Mineralogisch-Petrographisches Institut, Universität Hamburg, Germany; b Fachgebiet Kristallographie, FB Geowissenschaften, Universität Bremen, Germany; c MAPEX Center for Materials and Processes, Universität Bremen, Germany

**Keywords:** modulation, optic phonon, titanite, satellite reflections, DFPT

## Abstract

The crystal structure of the mineral malayaite has been studied by single-crystal X-ray diffraction at a temperature of 20 K and by calculation of its phonon dispersion using density functional perturbation theory. The X-ray diffraction data show first-order satellite diffraction maxima at positions **q** = 0.2606 (8)b*, that are absent at room temperature. The computed phonon dispersion indicates unstable modes associated with dynamic displacements of the Ca atoms. The largest-frequency modulus of these phonon instabilities is located close to a wavevector of **q** = 0.3b*. These results indicate that the malayaite crystal structure is incommensurately modulated by static displacement of the Ca atoms at low temperatures, caused by the softening of an optic phonon with *B_g_* symmetry.

## Abstract 2

The crystal structure of malayaite, CaSnOSiO_4_, at *T* = 20 K has been refined, based on the presence of satellite reflections with a modulation vector of 0.26b*. The structural modulation is attributed to a soft optic phonon, dominated by motion of the Ca atoms.

## 1. Introduction   

Malayaite is the tin analogue of the common accessory mineral titanite, CaTiOSiO_4_ (Takenouchi, 1976 ▸; Higgins & Ribbe, 1977 ▸). The crystal structure of malayaite at room temperature is described in space group *C*2/*c*.^1^ It consists of parallel, kinked chains of corner-sharing SnO_6_ octahedra, laterally connected by isolated SiO_4_ tetrahedra. In contrast to malayaite, pure titanite transforms from *C*2/*c* to *P*2_1_/*c* at temperatures below 490 K (Taylor & Brown, 1976 ▸), forming a crystal structure with ordered out-of-centre displacements of the Ti atoms inside their distorted octahedral coordination environment (Higgins & Ribbe, 1976 ▸; Speer & Gibbs, 1976 ▸).

With regard to the mechanism of phase transition and the nature of an intermediate polymorph between 490 and 825 K, the phase transition to the ordered titanite structure has been studied by numerous authors (Ghose *et al.*, 1991 ▸; Salje *et al.*, 1993 ▸; Zhang *et al.*, 1995 ▸; Kek *et al.*, 1997 ▸; Hayward *et al.*, 2000 ▸; Malcherek *et al.*, 2001 ▸; Malcherek, 2001 ▸). Analogous phase transitions also occur in structural analogues containing other *d* ^0^ transition elements (Malcherek *et al.*, 2004 ▸; Malcherek, 2007 ▸). The macroscopic formation of the ordered titanite structure is however suppressed in most natural titanite crystals which invariably contain impurity atoms such as Al or Fe (Higgins & Ribbe, 1976 ▸; Oberti *et al.*, 1991 ▸). One well known example of a cation substitution that suppresses the out-of-centre displacement of the octahedrally coordinated cation is that of Sn for Ti in the titanite–malayaite solid solution (Kunz *et al.*, 1997 ▸), which is a consequence of the absence of the second-order Jahn–Teller effect in the SnO_6_ octahedron (Kunz & Brown, 1995 ▸). When studying the computational prediction of the ordered titanite phase using density functional perturbation theory (DFPT), Malcherek & Fischer (2018 ▸) used malayaite as a reference system for the undistorted *C*2/*c* crystal structure. However, the calculated phonon dispersion of malayaite showed that several modes that are dominated by motion of the Ca atom are unstable at various wavevectors (Malcherek & Fischer, 2018 ▸). This prompted us to investigate the low-temperature crystal structure of malayaite in order to test whether any transition to a lower-symmetry structure occurs.

The Ca atom occupies interstices in the framework of corner-sharing SnO_6_ octahedra and SiO_4_ tetrahedra. At room temperature, the thermal displacement of Ca is strongly anisotropic (Higgins & Ribbe, 1977 ▸; Groat *et al.*, 1996 ▸), with its largest displacement amplitude extending subparallel to [001], *i.e.* the octahedral chain direction in the *C*2/*c* setting. Anomalies in the thermal expansion, in the temperature evolution of the Ca mean-square displacement as well as in the infrared and Raman spectra of malayaite have been observed near 500 K (Groat *et al.*, 1996 ▸; Meyer *et al.*, 1998 ▸; Zhang *et al.*, 1999 ▸), but with no obvious change in symmetry occurring at this temperature. Malayaite is further known to undergo a transition to triclinic symmetry at a pressure of 4.95 GPa, accompanied by an increase of the Ca coordination number from seven to eight (Rath *et al.*, 2003 ▸). Structural analogues of malayaite, such as CaGe_2_O_5_ or CaZrGeO_5_, exhibit temperature-driven monoclinic–triclinic polymorphism (Malcherek & Ellemann-Olesen, 2005 ▸). Even natural titanite, albeit Ta- and Al-rich, has been observed in triclinic symmetry (Lussier *et al.*, 2009 ▸), possibly driven by cation ordering. In the following we will describe another distortion of the malayaite structure that occurs at low temperatures and involves long-range modulation of the monoclinic malayaite structure.

## 2. Experimental and computational methods   

X-ray diffraction measurements of a natural single crystal of malayaite have been carried out at the P24 synchrotron beamline of PETRAIII/DESY in Hamburg, Germany. The temperature of the crystal was controlled using a Cryocool-LT He gas-stream cooler. Synchrotron radiation with λ = 0.61992 Å was obtained using a water-cooled Si double-crystal monochromator. Diffraction data were collected in ϕ and ω scans at two detector positions on a four-circle kappa diffractometer (EH1). Scattered X-rays have been detected using a MARCCD165 detector. Integration, reduction and correction of the scattering data were performed using *CrysAlisPRO* (Rigaku Oxford Diffraction, 2015 ▸). Because of ice formation, diffraction data arising from four of the ω scans have been omitted from the final low-temperature data set (Table 1 ▸). Structure refinement has been conducted using *Jana2006* (Petříček *et al.*, 2014 ▸).

The investigated malayaite crystal is from the El Hammam mine, Morocco (Sonnet & Verkaeren, 1989 ▸). The sample material has been characterized by electron microprobe analysis, yielding an average stoichiometry of Ca(Sn_0.97_Ti_0.03_)SiO_5_, with a very small, spatially inhomogeneous Ti^4+^ admixture.

First-principles calculations were performed by means of variational DFPT (Gonze, 1997 ▸; Gonze & Lee, 1997 ▸) as implemented in the CASTEP computer code (Clark *et al.*, 2005 ▸; Refson *et al.*, 2006 ▸). Details of these calculations are described by Malcherek & Fischer (2018 ▸). The plane-wave basis-set cut-off was set to 1200 eV. Norm-conserving pseudopotentials from the Bennett & Rappe pseudopotential library (Bennett, 2012 ▸), generated using the OPIUM code (Rappe *et al.*, 1990 ▸), have been employed. An irreducible set of 16 *k*-points in the Brillouin zone (BZ) has been sampled. The Monkhorst–Pack mesh was 4 × 4 × 3. Phonon calculations were conducted with the zero-pressure optimized crystal structure. Calculations for the base-centred lattice were carried out using the reduced cell with transformed cell parameters *a*_r_ = *b*_r_ ≠ *c*_r_ = *c*, α′ = β′ ≠ γ′.

In the following, results of these calculations are reported in the conventional *C*-centred setting. A 2 × 2 × 2 mesh of *q* vectors was used to calculate the phonon dispersion. The exchange-correlation (XC) energy contributions have been treated in the generalized gradient approximation (GGA) using the PBE and PBEsol functionals (Perdew *et al.*, 1996 ▸, 2008 ▸).

## 3. Results   

**Figure 1:**
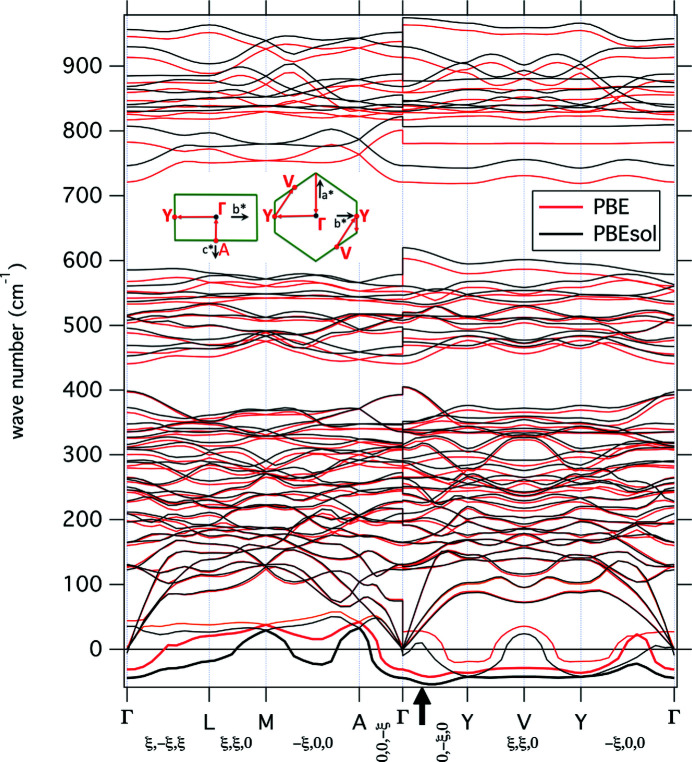
Calculated phonon dispersion of malayaite. Imaginary phonon frequencies of the unstable modes are shown in the real negative wavenumber range. The inset shows the location of special points and path details for the section A-Γ-Y-V-Y-Γ relative to the BZ boundary (green outline).

**Figure 2:**
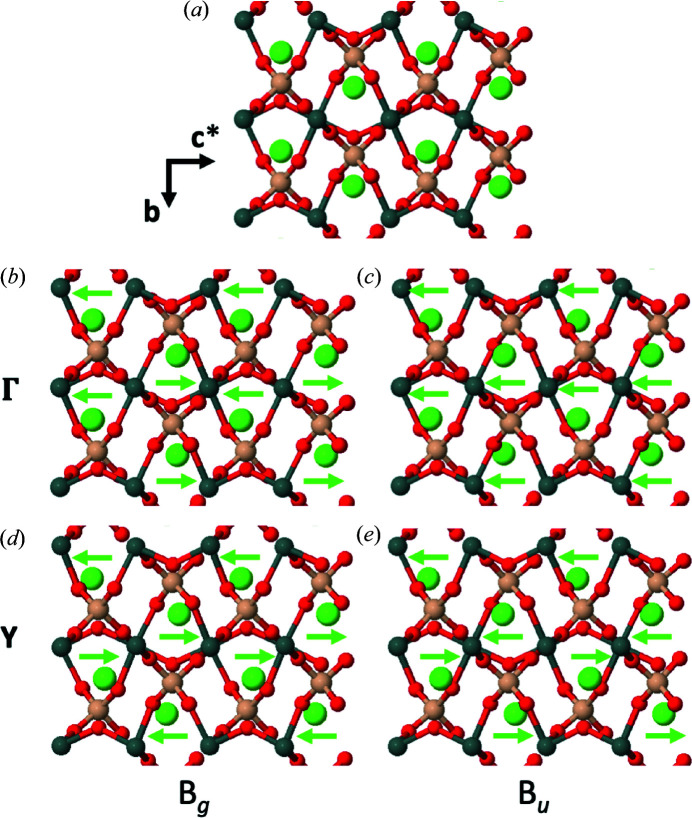
Crystal structure of malayaite in projection along [100] (*a*) and distortions caused by low- or imaginary-frequency *B_g_* and *B_u_* phonon modes (*b*)–(*e*). Green arrows indicate the relative motion of Ca atoms. The amplitude of the displacements is exaggerated for clarity. Si atoms are shown in light brown, Sn in grey, Ca in green and O in red.

Fig. 1 ▸ shows the calculated phonon dispersion of malayaite based on the two GGA approximations PBE and PBEsol. It is noteworthy that, unlike similar calculations made for titanite (Malcherek & Fischer, 2018 ▸), the two functionals yield very similar features, indicating that the results are not heavily dependent on the choice of functional. However, with PBEsol, especially frequencies in the Si–O stretching region above 800 cm^−1^ are systematically higher than frequencies obtained with the PBE approximation, due to the smaller volume overestimation calculated with PBEsol (Table 2 ▸). On the other hand, this tendency is reversed for the lowest calculated frequencies, where PBEsol yields systematically lower frequencies than PBE. Two at least partially unstable phonon mode branches are indicated in malayaite, plotted with negative frequencies in Fig. 1 ▸. Both modes are dominated by motion of the Ca atoms parallel to [001]. While some of the details for the lowest-frequency range seem to depend on the choice of XC functional, the Raman active *B_g_* mode has mostly imaginary frequency along the path section A-Γ-Y-V-Y-Γ, with the largest modulus located between Γ and Y, parallel to **b** ^*^ [*cf*. the inset of Fig. 1 ▸ and Malcherek & Fischer (2018 ▸) concerning path details] in both XC approximations. Compared with this, the infrared active *B_u_* mode is stable for most wavevectors, with the exception of those approaching the Y special point. This instability is enhanced with the PBEsol XC functional, which also predicts imaginary frequency for the transverse optical (TO) mode at the Γ point, due to larger LO/TO (LO = longitudinal optical) splitting compared with the PBE result. The type of motion associated with these two *B_g_* and *B_u_* modes is pictured in Fig. 2 ▸ for the Γ point, as well as for the BZ boundary point Y (0, 1, 0).

In light of the existence of a high-pressure phase transition to a triclinic malayaite polymorph and similar, but temperature-driven monoclinic to triclinic transitions in isostructural compounds, one plausible way to overcome the encountered dynamic instabilities is a structural transition to triclinic symmetry and space group 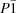, which is a maximal subgroup of *C*2/*c*. The unit cell of this triclinic structure is the reduced cell of the *C*2/*c* structure with possible distortions. DFPT calculations based on a malayaite crystal structure relaxed in triclinic symmetry did indeed render all phonon modes stable. However, the actual low-temperature crystal structure of malayaite turned out to be different, as shown in the following.

**Figure 3:**
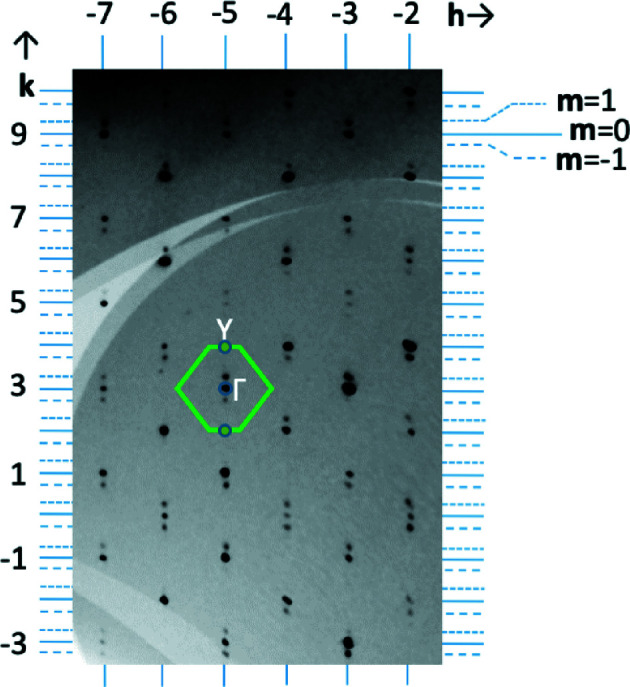
Reconstructed intensity in a section of the *hk*7*m* reciprocal-lattice plane, showing fundamental (*m* = 0) and first-order satellite diffraction maxima (*m* = 1, −1). The border of the first Brillouin zone of the *C*2/*c* parent structure is outlined in green at the 3,−5,7,0 reciprocal-lattice point.

**Figure 4:**
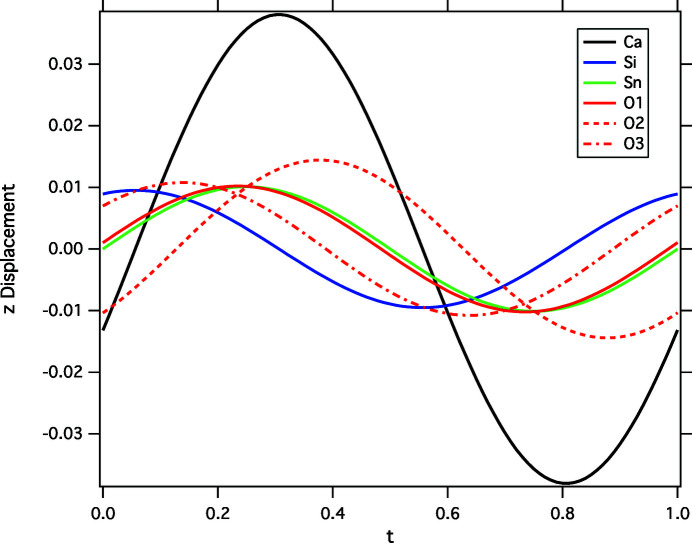
Atomic displacement along [001] as a function of the modulation coordinate *t*.

**Figure 5:**
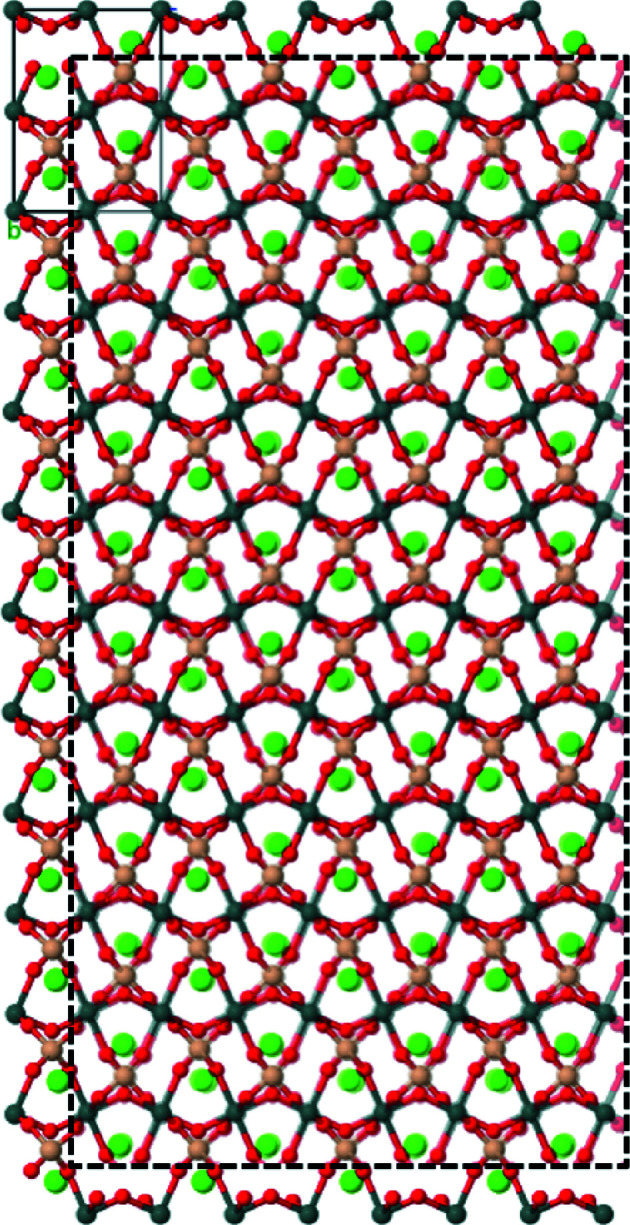
Superposition of the malayaite crystal structure in projection along [100], obtained as a snapshot of the structural distortions induced by the unstable *B_g_* phonon (transparent, framed foreground) and the modulated crystal structure refined against the 20 K diffraction data (background). Atomic displacements have been amplified by a factor of five for clarity. Compare Fig. 2 ▸ for orientation and colour coding.

Fig. 3 ▸ depicts a section of the *hk*7 layer of reciprocal space measured at a temperature of 20 K. Satellite reflections (*m* = 1, −1) with a modulation vector of **q** = 0.2606 (8)**b** ^*^ can be distinguished from the main reflections with *m* = 0 in this and other layers of reciprocal space. Only first-order satellite reflections are observed. The position of these satellites relative to the Γ and Y points of the first BZ (Fig. 3 ▸) is indeed close to the calculated shallow ‘minimum’ of imaginary phonon frequency of the lowest-frequency *B_g_* mode (indicated by an arrow in Fig. 1 ▸). That the calculated phonon instability matches the observed structure modulation rather well is further indicated by the fact that the Ca atoms of the refined modulated structure at 20 K do show the largest displacements, directed parallel to [001] (Fig. 4 ▸). The good agreement of these experimental observations with the dynamic structure modulations induced by the *B_g_* mode is demonstrated in Fig. 5 ▸, where the refined modulated structure is superimposed with a snapshot of the calculated phonon with wavevector (0, 0.3, 0), indicating a good match of both structure projections.

Thus the observed incommensurate structure modulation can be understood as a result of softening of the dynamic displacements associated with this phonon mode, *i.e.* static displacements of the atoms according to a single irreducible representation. The predicted instability of the phonon mode in the 0 K approximation supports the development of such static displacements at a certain, yet unknown critical temperature. The wavelength of the modulation at 20 K amounts to approximately 4*b*.

The resulting structure is described in the *C*2/*c*(0β0)*s*0 superspace group (de Wolff *et al.*, 1981 ▸; Janssen *et al.*, 2004 ▸). Using the parent space group and the modulation vector as input with the *Isosubgroup* utility (Stokes *et al.*, 2016 ▸, 2019 ▸), *C*2/*c*(0β0)*s*0 appears as a possible isotropy subgroup of *C*2/*c*, with LD2 as the active irreducible representation. The symmetry of malayaite remains monoclinic at 20 K. A few, low-intensity violations of the diffraction conditions for the *c*-glide plane appear above the 3σ(*I*) level in the *h*0*l* layer. These violations however appear irrespective of temperature and are likely caused by multiple scattering effects. No significant deviation from the monoclinic metric is observed.

Even at 20 K the Ca displacement tensor remains strongly anisotropic. The largest eigenvalue of the harmonic displacement tensor is about four times larger than the other two eigenvalues (0.0132, 0.0029 and 0.0030 Å^2^, respectively). This anisotropy is however smaller and more symmetric than for the room-temperature displacement tensor [0.0412, 0.0062 and 0.0037 Å^2^, also compare Higgins & Ribbe (1977 ▸)].

There is very little contraction of the unit-cell volume occurring down to 20 K (Table 2 ▸). *a* is almost constant and *b* even slightly expands with respect to the room-temperature value, while *c* contracts by a mere 0.0045 Å. The observed slight increase in the β angle continues the trend observed by Groat *et al.* (1996 ▸) at higher temperatures.

The Ca—O distances vary most strongly due to the structural modulation. This does predominantly affect the Ca—O3 distances subparallel to the octahedral chain direction (corresponding to [001] in the present setting), which vary between 2.7088 (6) and 2.7587 (6) Å, following the Ca displacement depicted in Fig. 4 ▸. The respective room-temperature bond distance is 2.7413 (5) Å.

**Figure 6:**
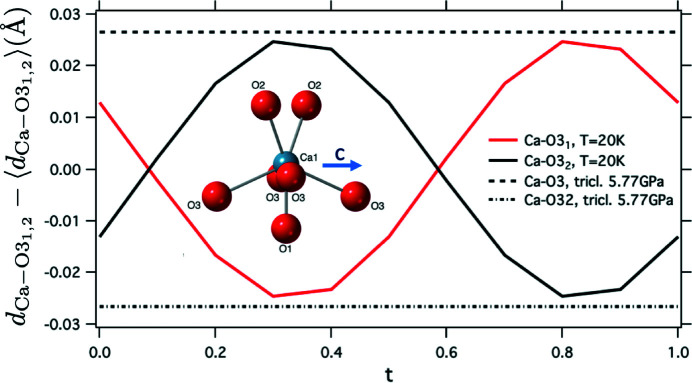
Deviation of bond distances Ca—O3_1, 2_ subparallel to [001] from their average, for the modulated crystal structure at *T* = 20 K (solid lines), compared with the corresponding deviation for the triclinic structure at 5.77 GPa (Rath *et al.*, 2003 ▸) (dashed lines). The inset shows the Ca coordination in projection parallel to **a** ^*^.

It is instructive to compare these Ca—O bond-length modulations with the Ca—O bond-length changes induced by the transition to triclinic symmetry observed at high-pressure conditions by Rath*et al.* (2003 ▸) (Fig. 6 ▸). At 5.77 GPa, the two Ca—O3 distances that are subparallel to the octahedral chain direction amount to 2.69 (1) and 2.743 (1) Å. The bond-length difference of 0.05 Å induced by the shift of the Ca atom is almost identical to the modulation range of the Ca—O3 bonds in the structure at 20 K. This emphasizes that the modulation described here and the triclinic distortion are induced by instability of the same type of atomic motion, with a finite wavevector (LD, Fig. 5 ▸) in the former and a zero wavevector [Γ, Fig. 2 ▸(*b*)] in the latter case.

The occurrence of the triclinically distorted structure in compounds of general composition Ca*M*O*X*O_4_ has been linked to a critical monoclinic distortion of the framework of octahedral chains and *X*O_4_ tetrahedra (Malcherek & Ellemann-Olesen, 2005 ▸). As the monoclinic β angle does not decrease with falling temperature (Table 2 ▸), such a critical monoclinic distortion is only attained in malayaite under high-pressure conditions, where the decrease of β below 113° correlates with the volume compression.

Based on bond-valence calculations with parameters taken from Brese & O’Keeffe (1991 ▸), the room-temperature structure of malayaite does exhibit overbonding of Sn and O1, due to a rather short Sn—O1 bond distance of 1.9470 (3) Å. This overbonding persists at 20 K, where the modulation however hardly affects the Sn—O1 bond distance.

## 4. Conclusions   

On the basis of the computational results, the crystal structure of malayaite at 20 K appears to be modulated by a soft *B_g_* optic phonon, leading to a transverse modulation of the Ca position with a period of close to 34 Å along [010]. The displacement is most pronounced along [001], *i.e.* the direction of the octahedral chains in the *C*2/*c* setting. While, to the best of the authors’ knowledge, no structure determination at similarly low temperatures has so far been conducted for titanite, it is unlikely that similar modulations occur in titanite, as the available phonon calculations for this compound (Gutmann *et al.*, 2013 ▸; Malcherek & Fischer, 2018 ▸) do not indicate any similar instabilities for the ordered *P*2_1_/*c* structure. The titanite phonon modes corresponding to the unstable modes of malayaite are stable in the GGA approximation, albeit at low frequency, even in the *C*2/*c* symmetry (Malcherek & Fischer, 2018 ▸). The second-order Jahn–Teller effect associated with the Ti atoms dominates in titanite, leading to the formation of a fully ordered structure that involves an ordered arrangement of short and long Ti—O bonds, modifying the position and dynamics of the Ca atoms in its wake. In malayaite this static distortion of the structural framework is absent and the monoclinic base-centred structure is retained to the lowest temperatures, with the lowest-frequency mode of the Ca atoms eventually softening to form the modulated structure. The exact temperature of the phase transition to this modulated structure remains to be determined.

## Supplementary Material


16372EQKyWN

Crystal structure: contains datablock(s) global, I, II. DOI: 10.1107/S2052520620003807/dk5091sup1.cif

Structure factors: contains datablock(s) I. DOI: 10.1107/S2052520620003807/dk5091Isup2.hkl

Structure factors: contains datablock(s) I. DOI: 10.1107/S2052520620003807/dk5091IIsup3.hkl

CCDC references: 1990518, 1990519

## Figures and Tables

**Table 1:** Experimental details

Crystal data		
Chemical formula	CaO_5_SiSn	CaO_5_SiSn
*M*_r_	266.87	266.87
Temperature (K)	20	298
Crystal system, space group	Monoclinic, 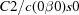	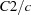
Wavevectors	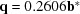	–
*a* (Å)	6.6663 (3)	6.6667 (2)
*b* (Å)	8.8954 (4)	8.8934 (3)
*c* (Å)	7.1475 (3)	7.1520 (3)
β (°)	113.405	113.323 (3)
*V* (Å^3^)	388.97 (3)	389.39 (3)
*Z*	4	4
Radiation type	X-ray, λ = 0.61992 Å	X-ray, λ = 0.61992 Å
μ (mm^−1^)	5.53	5.52
Crystal size (mm)	0.16 × 0.09 × 0.09	0.16 × 0.09 × 0.09
		
Data collection		
Diffractometer	Four-circle kappa	Four-circle kappa
Absorption correction	Multi-scan	Multi-scan
*T*_min_, *T*_max_	0.742, 1	0.754, 1
No. of measured, independent and observed [*I* > 3σ(*I*)] reflections	26 411, 7250, 5161	11 739, 2748, 2651
*R*_int_	0.031	0.019
(sinθ/λ)_max_ (Å^−1^)	1.251	1.251
		
Refinement		
*R* [*F* ^2^ > 3σ(*F* ^2^)], *wR* (*F* ^2^), *S*	0.044, 0.204, 1.30	0.026, 0.113, 1.06
*R* [*F* ^2^ > 3σ(*F* ^2^)], *wR* (*F* ^2^), *S* main reflections	0.039, 0.183	0.026, 0.113
*R* [*F* ^2^ > 3σ(*F* ^2^)], *wR* (*F* ^2^), *S* satellites	0.092, 0.26	-
No. of main reflections	2147	2651
No. of satellites	5101	0
No. of parameters	68	41
Δρ_max_, Δρ_min_ (e Å^−3^)	1.48, −2.04	0.7, −0.67

**Table 2:** Calculated and measured lattice parameters of malayaite

	*a* (Å)	*b* (Å)	*c* (Å)	β (°)	*V* (Å^3^)	*V*/*V*_298K_ − 1
PBE	6.8011	8.9864	7.3112	113.9	408.513	0.049
PBEsol	6.7095	8.9369	7.2291	113.69	396.96	0.019
Experimental, 298 K	6.6667 (1)	8.8934 (1)	7.1520 (1)	113.323 (1)	389.39 (1)	0
Experimental, 20 K	6.6663 (3)	8.8954 (4)	7.1475 (3)	113.405 (5)	388.97 (3)	−0.001

## Footnotes

1 Similar to titanite, literature descriptions of the structure of malayaite often use *International Tables for Crystallography* Vol. A setting *A*2/*a* (Aroyo *et al.*, 2006 ▸). Transformation to the default setting of space group 15 used in this work is achieved by a change of axis **c**, −**b**, **a** and an appropriate origin shift *e.g.* 0, ½, 0 in Inorganic Crystal Structure Database entry No. 1042.
